# Inhibition of CAL27 Oral Squamous Carcinoma Cell by Targeting Hedgehog Pathway With Vismodegib or Itraconazole

**DOI:** 10.3389/fonc.2020.563838

**Published:** 2020-11-10

**Authors:** Raíza Dias Freitas, Rosane Borges Dias, Manuela Torres Andion Vidal, Ludmila de Faro Valverde, Rafaela Gomes Alves Costa, Andresa Karen Andrade Damasceno, Caroline Brandi Schlaepfer Sales, Leonardo de Oliveira Siquara da Rocha, Mitermayer Galvão dos Reis, Milena Botelho Pereira Soares, Ricardo Della Coletta, Thiago Almeida Pereira, Daniel Pereira Bezerra, Clarissa Araújo Gurgel Rocha

**Affiliations:** ^1^ Gonçalo Moniz Institute, Oswaldo Cruz Foundation (IGM-FIOCRUZ/BA), Salvador, Brazil; ^2^ Department of Pathology and Forensic Medicine, School of Medicine of the Federal University of Bahia, Salvador, Brazil; ^3^ Department of Propaedeutics, School of Dentistry of the Federal University of Bahia, Bahia, Brazil; ^4^ Department of Biomorphology, Institute of Health Sciences, Federal University of Bahia, Salvador, Brazil; ^5^ Department of Oral Diagnostics, School of Dentistry, University of Campinas, Piracicaba, Brazil; ^6^ Institute for Stem Cell Biology and Regenerative Medicine, Stanford University, Stanford, CA, United States

**Keywords:** oral squamous cell carcinoma, hedgehog pathway, vismodegib, itraconazole, real-time polymerase chain reaction

## Abstract

Oral Squamous Cell Carcinoma (OSCC) presents an important challenge for the health systems worldwide. Thus, unraveling the biological mechanisms involved in OSCC pathogenesis is essential to the discovery of new drugs with anticancer potential. The Hedgehog (HH) pathway has shown promising results as a therapeutic target both *in vitro* and *in vivo.* This study aimed to investigate the effects of vismodegib and itraconazole on the expression of Hedgehog (HH) genes (PTCH1, SMO, and GLI1), cell cycle and cell death in OSCC cells. Alamar Blue assay was used to assess the cytotoxicity of vismodegib and itraconazole in a panel of oral cancer cell lines, including CAL27. The expression of HH signaling components after treatment with vismodegib and itraconazole, at concentrations of 25 or 50 μg/ml was evaluated by qPCR. Cell cycle and apoptosis were evaluated by flow cytometry after 72 h treatment with 50 μg/ml of vismodegib or itraconazole. HH signaling was activated in OSCC cell lines CAL27, SCC4, SCC9, and HSC3. Vismodegib and itraconazole significantly reduced CAL27 cell viability after 48 h of treatment. Gene expression of PTCH1, SMO, and GLI1 decreased in response to 24 h of treatment with vismodegib or itraconazole. Furthermore, CAL27 cells exhibited alterations in morphology, cell size, and cellular granularity. An increase in the DNA fragmentation was observed after treatment and both inhibitors induced apoptosis after 72 h. In conclusion, SMO inhibitors vismodegib and itraconazole demonstrably reduced the expression of HH genes in CAL27 OSCC cell line. In addition, treatment with vismodegib and itraconazole reduced cellular viability and altered the morphology of CAL27 cells, and also induced apoptosis.

## Introduction

Head and neck cancers pose a serious public health problem due to its high incidence, prevalence, and mortality (1–3). Among these, cancers affecting the oral cavity rank in the top 20 of all malignant tumors worldwide, with more than 350,000 new cases reported in 2018 (4). Oral squamous cell carcinoma (OSCC) is the most frequently histological type, corresponding to 90% of all diagnoses (5–7). According to the classification criteria for oral cavity and oropharynx tumors established by the World Health Organization (8), OSCC is considered an invasive and aggressive epithelial neoplasia with the potential to promote early metastasis and extensive lymph node involvement. This disease mainly affects adult population aged 50 to 60 years old and has a multifactorial etiology, with the principal risk factors being tobacco and alcohol use (9–12). A 5-year survival rate has been reported in approximately 50% of OSCC cases (3, 13).

Despite advances in cancer treatment, the therapeutic options for OSSC remain limited, with surgery and radiotherapy being the most common approaches. The lack of robust treatment options has led to high morbidity, reduced quality of life, and a high frequency of metastatic disease (14). Accordingly, obtaining a comprehensive understanding of biology of this type of tumor is crucial to the development of new therapeutic strategies. Advances in knowledge will also improve the clinical outcome and survival of OSCC patients, since morbidities associated with surgical treatment (e.g. facial deformity, speech problems, difficulty swallowing, etc.) constitute an important aspect of this disease, leading to social, emotional, and economic impacts (e.g. low productivity at work, difficulty in re-entering the labor market, etc.).

Our group was one of the pioneers in demonstrating that the Hedgehog (HH) cascade becomes reactivated in OSCC (15). Considering that, this pathway becomes dysregulated in several types of carcinomas, such as lung (16–18), breast (19, 20), ovaries (21, 22), and liver (23, 24), HH molecules can be considered as potential pharmacological targets in OSCC. Of special interest in the HH pathway are the Sonic ligand (Shh), the Patched receptor (PTCH1), the Smoothened co-receptor (SMO), and GLI transcription factors, which are the major components of the HH pathway responsible for cellular proliferation, self-renewal, migration, and stem cell-population maintenance, among others (25–28). In brief, this cascade is activated by binding one of secreted ligands (Shh, Ihh, or Dhh) to the PTCH1 or PTCH2 receptor expressed by responsive cells. PTCH1-ligand complex promotes conformational changes in SMO, resulting in its activation, signal transduction into cytoplasm and, consequently, to the activation and nuclear translocation of GLI transcription factors, which will ultimately activate target genes (27, 29). HH cascade activation may also occur due to activating mutations in SMO or inactivating mutations in PTCH1 (20, 30).

The search for drugs capable of blocking the HH pathway has already produced important results. Vismodegib (GDC-0449) was the first HH signaling pathway targeting agent approved by the US Food and Drug Administration (FDA) and European Medicines Agency (EMA) for the treatment of advanced local or metastatic basal cell carcinomas in patients who are not suitable candidates for surgical resection or radiotherapy (31–35). Many clinical studies have investigated the effects of vismodegib, either as monotherapy or in combination with other drugs, on various types of cancers, such as advanced chondrosarcoma (NCT01267955) (36), metastatic breast cancer (NCT01071564) (37), medulloblastoma (NCT00939484) (38), sarcoma (NCT01154452) (39), and others (US, NLM, 2018).

Several mechanisms of resistance to vismodegib have been demonstrated in studies using medulloblastoma and basal cell carcinoma cells (40, 41), which may be related to its pharmacokinetic properties, including high affinity for plasma proteins, limited absorption, and slow elimination (42, 43), or related to GLI1 activation, which can be mediated by other signaling pathways (44–46).

Itraconazole, a broad-spectrum antifungal agent that inhibits lanosterol 14-α-demethylase (14LDM), an enzyme that produces ergosterol in fungi and cholesterol in mammals (4–8), is also a known regulator of the HH pathway (47–49). This compound prevents the accumulation of SMO in the primary cilia (47), and therefore stands out as a potential agent for combating the treatment resistance seen in other anti-HH compounds, such as vismodegib. Moreover, several clinical trials are currently underway to investigate the inhibitory potential of itraconazole in various cancer types, including esophageal (NCT02749513) (50), ovarian (NCT03081702) (51), non-small cell lung (NCT02357836) (52), and others (US, NLM, 2018).

Despite the promising results seen in several types of cancer using vismodegib and itraconazole (34, 53), their therapeutic potential of blocking the HH pathway in OSCC has not been studied. The present study attempted to demonstrate the *in vitro* effects of vismodegib and itraconazole on the expression of HH pathway genes, as well as OSCC cell proliferation and death.

## Materials and Methods

### Cell Culturing

All human cell lines (**Table S1**) were cultured in cell culture flasks (75 cm^3^, 250 ml volume) in DMEM medium (Life Technologies, Gibco^®^; Carlsbad, CA, USA) supplemented with 10% fetal bovine serum (FBS, Life Technologies, Gibco^®^; Carlsbad, CA, USA) and 50 μg/ml gentamicin (Novafarma, Anapolis, GO, Brazil). Cultures were maintained in incubators under 5% CO_2_ at 37°C and monitored daily using an inverted microscope. Cell dissociation with trypsin was performed when cell growth reached the confluence of 70 to 80% of the total culture flask volume. Cell lines were tested monthly for mycoplasma contamination using Hoechst dye (Sigma-Aldrich; St Louis, USA).

#### PBMC Preparation

Human peripheral blood mononuclear cells (PBMC) were obtained from the peripheral blood of healthy non-smokers aged 25–35 years who had no reported drug or medication use for at least 15 days prior to collection. The Institutional Review Board of the Oswaldo Cruz Foundation (FIOCRUZ, Salvador, Bahia, Brazil) approved the present experimental protocol (Number 031019/2013). All participants signed a term of informed consent to participate in the study. Blood collection (up to a final volume of 5 ml) was performed in heparinized flasks by trained professionals at Fiocruz using sterile disposable syringes.

PBMCs were isolated following a standard protocol by centrifugation using a Ficoll density gradient (Ficoll-Paque Plus; GE Healthcare Bio-Sciences AB; Chicago, IL, USA). After separation, cells were washed twice with saline, resuspended (0.3 × 10^6^ cells/ml) in RPMI medium supplemented with 20% FBS, 2 mM glutamine, and 50 μg/ml gentamicin. To induce T cell proliferation, 10 μg/ml concanavalin A (Con A; Sigma Chemical Co; St Louis, MO, USA.) was added for use as a mitogen.

### Gene Expression of HH Pathway Components in OSCC

To characterize the expression of the studied HH pathway components (SHH, PTCH1, SMO, and GLI1), OSCCs were maintained under serum-free condition for 24 h, since FBS is known to inhibit the expression of HH molecules, as previously reported (54).

#### Total RNA Isolation and Reverse Transcription (RT-PCR)

For total RNA isolation, OSCC cells were plated on 6-well plates at a density of 0.7 × 10^5^ cells/ml per well in 2.5 ml of complete medium. After, 24 and 48 h cells were directly collected to the buffer lysis solution (RLT, Rneasy^®^ Mini Kit, QIAGEN; Hilden, Germany). RNA was extracted using silica microcolumns (Rneasy^®^ Mini Kit, QIAGEN; Hilden, Germany) and eluted in 20 µl of water. The quantity and purity of the RNA preparations was analyzed using Qubit™ RNA Assay Kit (Thermo Fisher Scientific, USA) in a fluorometer (QuBit™, Life Technologies; Carlsbad, CA, USA). Reverse transcription was performed using the Superscript VILO™ master mix (Invitrogen Corporation, USA) after elimination of genomic DNA with DNase I, Amplification Grade (Invitrogen Corporation, USA), during 10 min. All resulting cDNA samples were stored at −20°C. Experiments were performed under DNAse/RNAse-free conditions.

#### HH Pathway Gene Expression

HH pathway component expression was evaluated by qPCR using inventoried TaqMan Gene Expression Assays™ for genes SHH (Hs00179843_m1), PTCH1 (Hs00181117_m1), SMO (Hs01090242_m1), and GLI1 (Hs01110766_m1), as well as for the reference gene B2M (Hs99999907_m1). Reactions were run on an ABI ViiA7 system (Applied Biosystems™; Foster City, CA, USA) using a 96 Fast Well Block with total volumes of 20 μl containing 1 µg of total RNA. The amplification process consisted of an initial step at 50°C for 2 min, followed by 95°C for 10 min, then 40 cycles at 95°C for 15 s and 60°C for 1 min.

After the amplification and dissociation runs, Quantification Cycle (Cq) values ​​were obtained using the Expression Suite software (Applied Biosystems; Foster City, CA, USA). For the comparison of experimental groups and controls by relative quantification, the Cq comparative method (2^-ΔΔCQ^) was used. The B2M reference gene was used as a normalizer (55) and reactions were calibrated using the Cq values ​​of samples treated with dimethylsulfoxide (DMSO 0.2%) as a negative control. All qPCR reactions were performed in three independent experiments with duplicate samples.

### Assessment of Cytotoxicity by Alamar Blue Assay

To assess the cytotoxicity on tumor and non-tumor cells, Alamar blue assay was performed following treatment, as described by Ahmed et al. (56). Tumor and non-tumor cells were distributed on 96-well plates at a density of 0.7 × 10^5^ cells/ml in 100 μl of complete medium.

Following dissolution in 0.5% DMSO, each inhibitor was added and the plates were incubated for 72 h at concentrations ranging from 0.39 to 50 µg/ml. Doxorubicin and 5-fluorouracil were used as positive controls at concentrations ranging from 0.03 to 5 µg/ml. Cells treated with the vehicle (DMSO 0.5%) used to dilute the tested inhibitors were employed as a negative control. After 68 or 48 h (PBMC) of incubation, 20 μl of Alamar blue stock solution (0.312 mg/ml) was added to each well. Absorbance was measured at wavelengths of 570 nm (reduced) and 600 nm (oxidized) using a microplate reader (Molecular Devices; Sunnyvale, CA, USA).

The CAL27 cells were selected for use in all following *in vitro* assays, which allowed us to better evaluate the effects of vismodegib and itraconazole on HH pathway component gene expression, as well as cell viability, cycle, and death patterns. Additionally, Alamar blue assays were also performed in CAL27 cells following treatment of 6, 12, 24, 48, and 72 h to confirm the cell response to SMO inhibitors. After determining the IC50 value for SMO inhibitors, all following assays were performed using two concentrations (25 or 50 µg/ml), in CAL27 cells. All results were confirmed by at least three independent experiments with duplicate samples.

### SMO Inhibitor Concentrations and Specifications

For all inhibition assays, the test compounds vismodegib (Selleck Chemicals; Houston, USA) and itraconazole (Sigma-Aldrich; St. Louis, MO, USA) were used. Doxorubicin (IMA S.A.I.C. Lab; Buenos Aires, Argentina) and 5-fluorouracil (5-FU) (Sigma-Aldrich; St Louis, MO, USA) were used as positive controls, as both chemotherapeutic agents are approved by the FDA for the treatment of several cancers, including breast, pancreatic, ovarian, colon, and stomach (NIH, 2017). All compounds used in experimentation were dissolved in 0.5% DMSO and diluted in saline to variable concentrations in accordance with each assay performed (**Table S2**). These concentrations were determined using the IC_50_ values obtained for each compound in each evaluated cell line. To evaluate any possible effects of the drug vehicle, some assays included 0.5% DMSO as a control.

### Cell Viability Assay—Trypan Blue

To assess the viability of CAL27 cells, a trypan blue assay was performed after 24 and 48 h treatment. OSCC cells plated on 6-well plates at a density of 0.7 × 10^5^ cells/ml at a final volume of 2.5 ml were incubated with vismodegib and itraconazole for 24 and 48 h. For this analysis, 90 μl was removed from the plate using trypsin, stained with 10 μl trypan blue and counted in a Neubauer chamber, under optical microscopy, at 20× magnification (Olympus CX41, Olympus, Tokyo, Japan). All results were confirmed by at least three independent experiments.

### Morphological Evaluation

For morphological analysis, CAL27 cells were incubated on 6-well culture plates at a density of 0.7 × 10^5^ cells/ml at a final volume of 2.5 ml containing the SMO inhibitors. Cells were observed both before and after treatment using an inverted microscope (DMi8, Leica; Wetzlar, Germany). Digital images were captured using Leica software Application Suite X (XLAS X, Leica Microsystems; Wetzlar, Germany) for the analysis of morphological changes. Additionally, flow cytometry was performed to evaluate FSC (forward scatter) and SSC (side scatter) standards to demonstrate relative size and granularity or internal cell complexity, respectively.

### Cell Cycle and Cell Death Analyses

The effects of vismodegib and itraconazole on cell cycle and cell death were analyzed using the CAL27 cell line. In all experiments, 2.5 ml of cell suspension (0.7 × 10^5^ cells/ml) was plated into each well of 6-well plates and incubated overnight to allow cell adhesion, followed by a 72 h treatment period with vismodegib and itraconazole. Doxorubicin and 5-FU were used as positive controls. Flow cytometry was performed on a BD LSRFortessa cytometer using the BD FACSDiva Software (BD Biosciences; Franklin Lakes, NJ, USA) and Flowjo Software 10 (Flowjo LCC; Ashland, OR, USA). Experiments were performed in duplicate and repeated at least three times.

#### Analysis of DNA Fragmentation and Cell Cycle With Propidium Iodide

Nuclear DNA content, which reflects the cell cycle phase, was evaluated by flow cytometry using propidium iodide (PI) as a fluorogenic agent. After 72 h treatment with the test compounds, cells were treated with a permeabilization solution (300 μl) containing 0.1% triton X-100, 0.1% sodium citrate, 2 μg/ml PI, and 100 μg/ml RNase in distilled water, in the absence of light and at room temperature. After 30 min, cell fluorescence was measured by flow cytometry on a LSRFortessa cytometer using FACSDiva Software version 6.2 (Becton Dickinson Biosciences; San Jose, CA, USA). Proportions of fragmented internucleosomal DNA and cell cycle phases were determined using Flowjo software, v 10 (Flowjo LCC; Ashland, OR, USA). Cells in the sub-G1 phase represent internucleosomal DNA fragmentation. Cellular debris were omitted from the analysis and 10,000 events were analyzed per sample.

#### Cell Death Pattern Analysis Using Annexin V and Propidium Iodide

After 48 and 72 h of treatment with the HH pathway inhibitors, cells were stained with annexin V-FITC and PI (BD Biosciences; Franklin Lakes, NJ, USA) to assess cell viability (categories: viable - Q4, early apoptosis - Q3, late apoptosis - Q2, or necrotic - Q1.) Cell fluorescence was measured by flow cytometry, as above.

### Statistical Analyses

Statistical analysis was performed using the GraphPad Prism program. Data were analyzed according to distribution using a normal Gaussian curve. IC_50_ values ​​were obtained by non-linear regression, considering three independent experiments performed in duplicate. Differences between groups were evaluated by ANOVA (analysis of variance), followed by the Student-Newman-Keuls test (p < 0.05).

Regarding gene expression analysis, relative quantification (QR) values ​​were obtained with the aid of the Gene Expression Suite™ program (Applied Biosystems) according to the Cq comparative method (ΔΔ CQ) (57).

## Results

### The HH Pathway Is Activated in Human Oral Cancer Cell Lines

Initially, we investigated the transcriptional activity of HH components by qPCR. HH pathway activity was confirmed *via* the constitutive expression of GLI-1 gene, the gold standard for determining HH pathway activation (58–60), in all OSCC cell lines tested. The SHH ligand was constitutively expressed in CAL27 and SCC4 cell lines (**Figure S1**).

### Cytotoxic Activity of Vismodegib and Itraconazole in OSCC Cell Lines

To evaluate the tumor cytotoxicity of the SMO inhibitors vismodegib and itraconazole, a cytotoxicity assay was performed using Alamar Blue after 72 h of treatment. Vismodegib presented cytotoxicity, with IC_50_ values ​​ranging from 34.03 μg/ml in CAL27 and 41.30 in HSC-3. IC_­50_­ values ​​for itraconazole were similar for all cell lines tested (**Table 1**). The positive controls doxorubicin and 5-FU demonstrated cytotoxic activity (<4μg/ml) in all tumor lines. The treatment and positive-control and compounds were also evaluated in two non-tumor cell types, HaCaT, an immortalized non-transformed keratinocyte cell line, and in a primary culture of peripheral blood mononuclear cells (PBMC). Vismodegib presented an IC_­50_­ of 23.81 μg/ml in HaCaT and 31.16 μg/ml in PBMCs, whereas itraconazole presented IC­_50_ values ​​of 17.66 μg/ml and 15.35 μg/ml in HaCaT and PBMC, respectively (**Table 1**).

**Table 1:** Human tumor lineages used in the Alamar Blue cytotoxicity assay.

Cells	IC_50_ in μg/ml			
	DOX	5-FU	Vismodegib	Itraconazole
CAL27	1.68 1.21–2.34	5.1 3.7–7.2	34.03 29.1–39.9	>50
HSC 3	0.19 0.05–0.66	2.18 1.37–3.47	41.30 25.26–67.53	>50
SCC 4	0.04 0.03–0.06	N.d.	35.03 25.96–47.29	49.03 35.17–68.35
HaCaT	0.06 0.01–0.30	N.d.	23.81 4.85–116.7	17.66 0.78–399.4
PBMC	2.81 1.39–5.67	>25	31.16 15.02–64.68	15.35 3.68–63.96

Data presented as IC_­50_­ values in μg/ml with a 95% confidence interval obtained by nonlinear regression from at least three independent experiments performed in duplicate. Cytotoxicity measured by Alamar Blue assay, 72 h after treatment. Doxorubicin (DOX) and 5-FU were used as positive controls. N.d., not determined.

The CAL27 cell line was selected to determine the optimal treatment time for vismodegib and itraconazole. Cytotoxicity was evaluated at the 6, 12, 24, 48, and 72 h time points. At 6 and 12 h, IC_­50_ values of 5-FU and the test compounds were unable to be determined. However, after 24, and at 48 and 72 h of treatment, vismodegib presented IC_­50_ values of 54.9, 41.5, and 34.03 μg/ml, respectively. It was not possible to determine IC_50_ values for itraconazole at any of the time points evaluated. The IC­_50_ values ​​of 5-FU were 95.6, 28.2 and 5.1 μg/ml at 24, 48 and 72 h of treatment, respectively (**Table 2**).

**Table 2:** Cytotoxic activity of 5-FU, vismodegib, and itraconazole in the CAL-27 cell line.

Treatment time	IC_50_ in μg/ml		
	5-FU	Itraconazole	Vismodegib
6 h	>25	>50	>50
12 h	>25	>50	>50
24 h	95.6 (35.6–257.0)	>50	54.9 (45.5–66.3)
48 h	28.2 (12.4–64.0)	>50	41.5 (34.4–50.2)
72 h	5.1 (3.7–7.2)	>50	34.03 (29.1–39.9)

Data presented as IC_­50_­ values in μg/ml with a 95% confidence interval obtained by nonlinear regression from at least three independent experiments performed in duplicate. Cytotoxicity measured by Alamar Blue assay, 6, 12, 24, 48, and 72 h after treatment. 5-FU was used as positive control.

### Vismodegib and Itraconazole Reduce the Viability of CAL27 Cell Line

**Figure 1:**
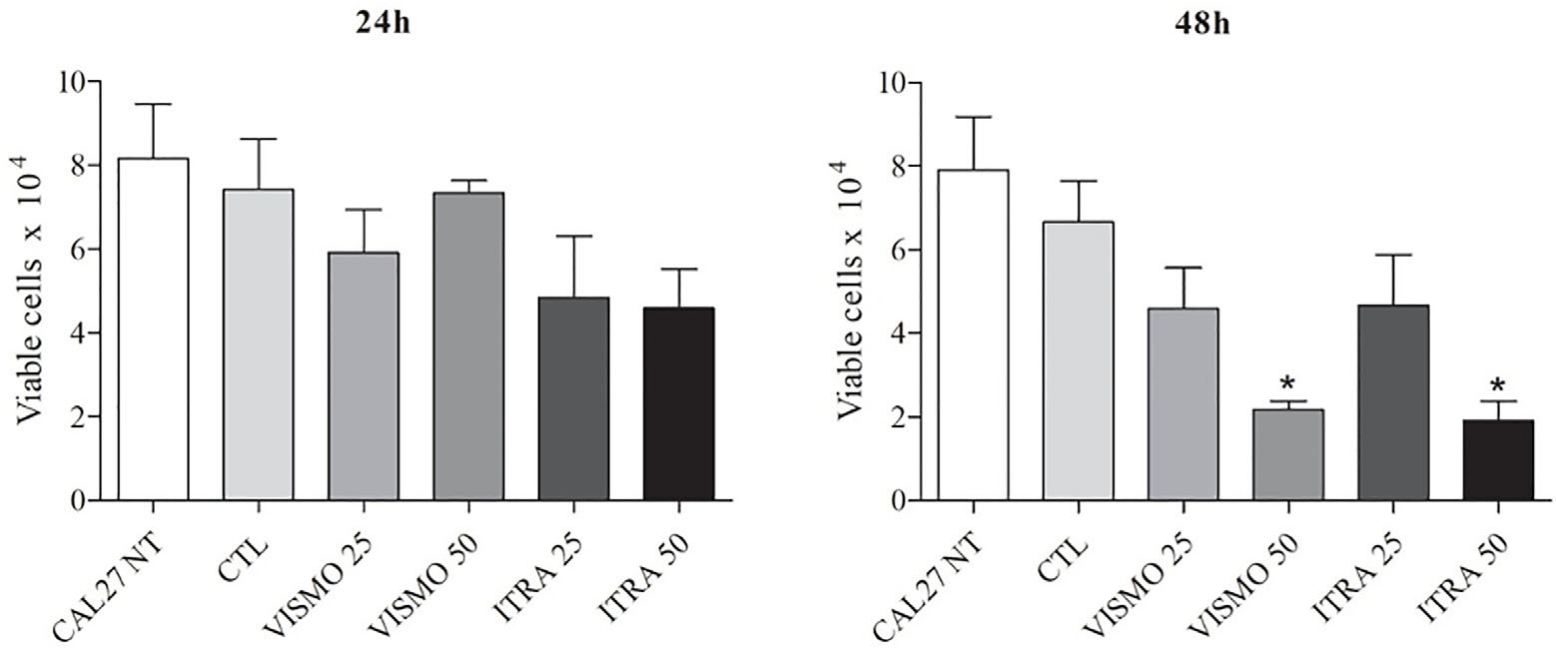
Effect of SMO inhibitors on CAL27 cell line viability. CAL27 cells were cultured for 24 or 48 h in the absence (CAL27 NT), or presence of vismodegib (VISMO) or itraconazole (ITRA). One negative control (CTL) was treated with the vehicle (0.2% DMSO) used to solubilize test compounds. Cell viability was determined using a trypan blue dye exclusion assay. Values ​​represent mean ± S.E.M. from three independent experiments performed in duplicate. *p < 0.05 compared to the CTL group by ANOVA, followed by the Student Newman-Keuls test.

Vismodegib is a small-molecule inhibitor of Smoothened (34, 35), while itraconazole reduces HH pathway activity by inhibiting the accumulation of SMO in primary cilium (45). We first examined the effects of vismodegib on the viability of CAL27 OSCC cell line. At 24 h, the greatest effect on cell viability was seen using itraconazole at 50 μg/ml, followed by itraconazole at 25 μg/ml. At 48 h, significant reductions in the number of viable cells were observed after treatment with itraconazole and vismodegib at 50 μg/ml in comparison to untreated and vehicle-treated groups (p < 0.05) (**Figure 1**).

### Treatment With SMO Inhibitors Altered CAL27 Cell Morphology, Volume, and Granularity

Analysis of cell populations showed morphology alterations in cells treated with vismodegib or itraconazole at 24 or 48 h, compared to the untreated and vehicle groups (**Figures S2**, **S3**, **S4**, and **S5**). With regard to morphology, an evident aspect visualized in the treatment groups was reduced numbers and volumes of tumor islands, with cells exhibiting greater loss of adhesion and nuclear fading (**Figures S2**, **S3**, **S4**, and **S5**). Cellular retraction was also observed, especially in cells treated for 48 h with itraconazole (**Figures S4** and **S5**).

**Figure 2:**
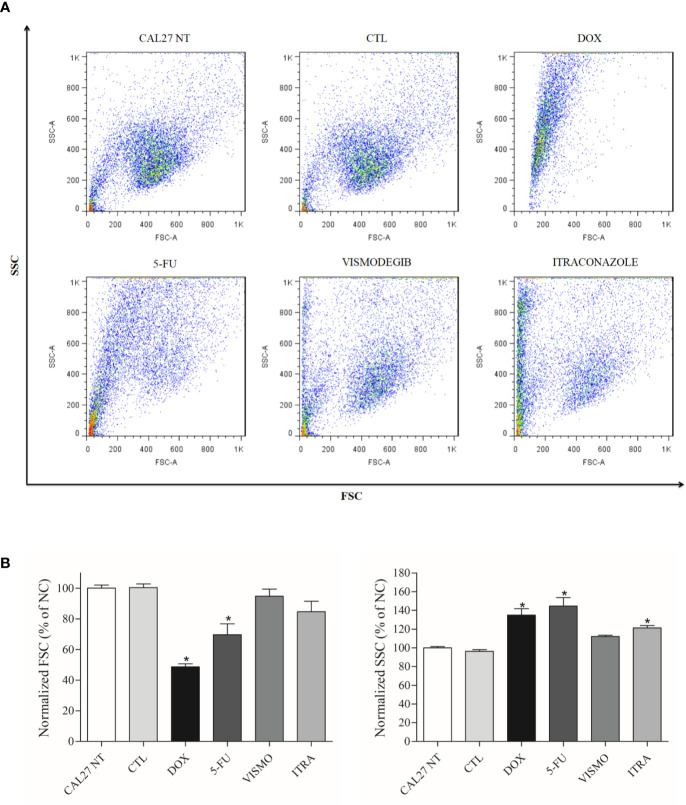
**(A)** Scatterplots and **(B)** Graphical representation of light scattering characteristics of CAL27 cells treated with vismodegib and itraconazole, as determined by flow cytometry after 72 h of treatment. FSC (forward scatter) and SSC (side scatter) were used to demonstrate relative size and granularity or internal cell complexity. FSC and SSC values were normalized with the negative control (DMSO 0.2%). The negative control was treated with the vehicle (DMSO 0.2%) used to solubilize and dilute the compounds. Doxorubicin (DOX, 1 μg/ml) and 5-FU (10 μg/ml) were used as positive controls. Data is representative of three independent experiments performed in duplicate. Cellular debris was omitted from the analyses and 10,000 events were analyzed per sample. *p < 0.05 compared to the CTL group by ANOVA, followed by the Student Newman-Keuls test.

At only after 72 h, flow cytometry analysis revealed cellular retraction arising from treatment with the SMO inhibitors, as evidenced by a decrease in Forward Light Scatter (FSC), and nuclear condensation due to increased lateral dispersion (SSC), as demonstrated by the scatterplots in **Figure 2A**. The positive controls doxorubicin and 5-FU were found to decrease cell size (p < 0.05). In addition, these two controls and itraconazole at 50 μg/ml were found to increase the granularity of CAL27 cells (p < 0.05) (**Figure 2**).

### Treatment With Vismodegib or Itraconazole Reduced the Expression of SMO, PTCH1, and GLI1

**Figure 3:**
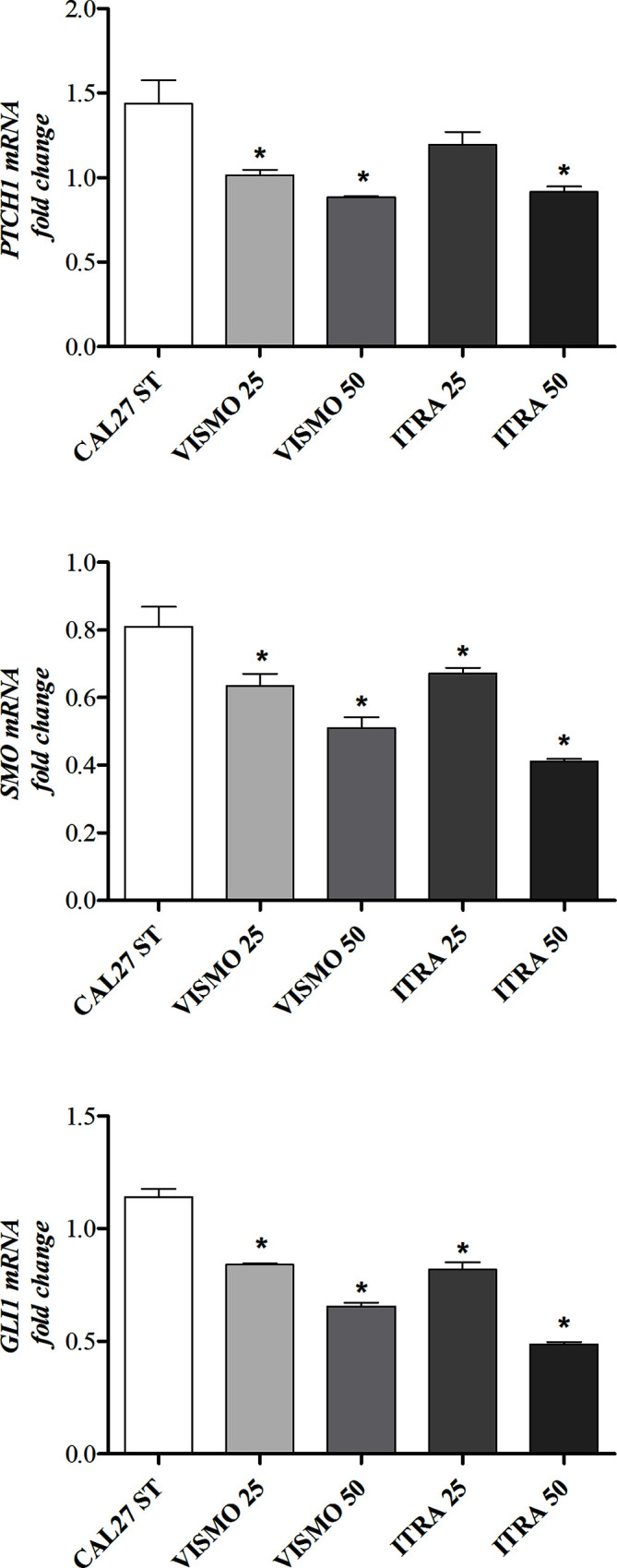
HH pathway component gene expression profiles after 24 h of treatment with vismodegib or itraconazole (at 25 or 50 µg/ml) in CAL27 cells. Negative control was treated with DMSO (0.2%), used to solubilize and dilute tested compounds. Doxorubicin (DOX, 1 μg/ml) and 5-FU (10 μg/ml) were used as positive controls. The value of relative quantification (RQ) used in each sample was normalized using the B2M reference gene and calibrated according to RQ values obtained for the CAL27 treated with DMSO (0.2%). qPCR reactions were performed in cells treated and non-treated with SMO inhibitors. *p < 0.05 compared to the CTL group by ANOVA, followed by the Student Newman-Keuls test.

After 24 h of treatment with vismodegib or itraconazole (at 25 or 50 µg/ml), reduced gene expression of SMO, PTCH1, and GLI1 were observed (**Figure 3**).

### Vismodegib and Itraconazole Induce Apoptosis in CAL27 Cells

**Figure 4:**
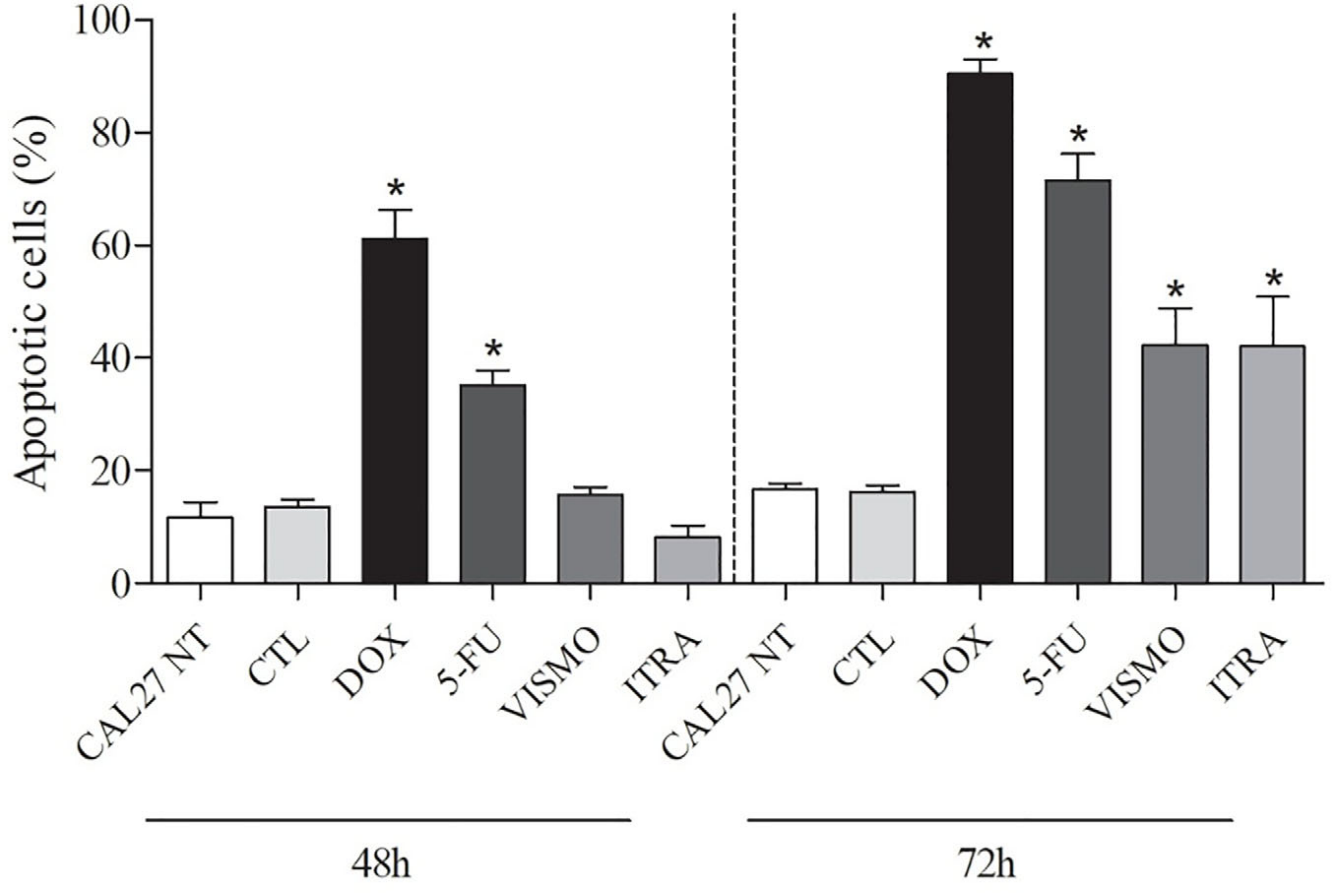
Effects of vismodegib and itraconazole on the externalization of phosphatidylserine in CAL27 cells, as determined by flow cytometry using annexin V-FITC after 48 and 72 h of treatment. Negative control was treated with the vehicle (0.2% DMSO) used to solubilize and dilute test compounds. Doxorubicin (DOX, 1 μg/ml) and 5-FU (10 μg/ml) were used as positive controls. Values ​​are expressed as means ± S.E.M. from three independent experiments performed in duplicate. Cellular debris was omitted from analyzes and 10,000 events were analyzed per sample. *p < 0.05 when compared to negative controls by ANOVA (analysis of variance) followed by the Student Newman-Keuls test.

**Figure 5:**
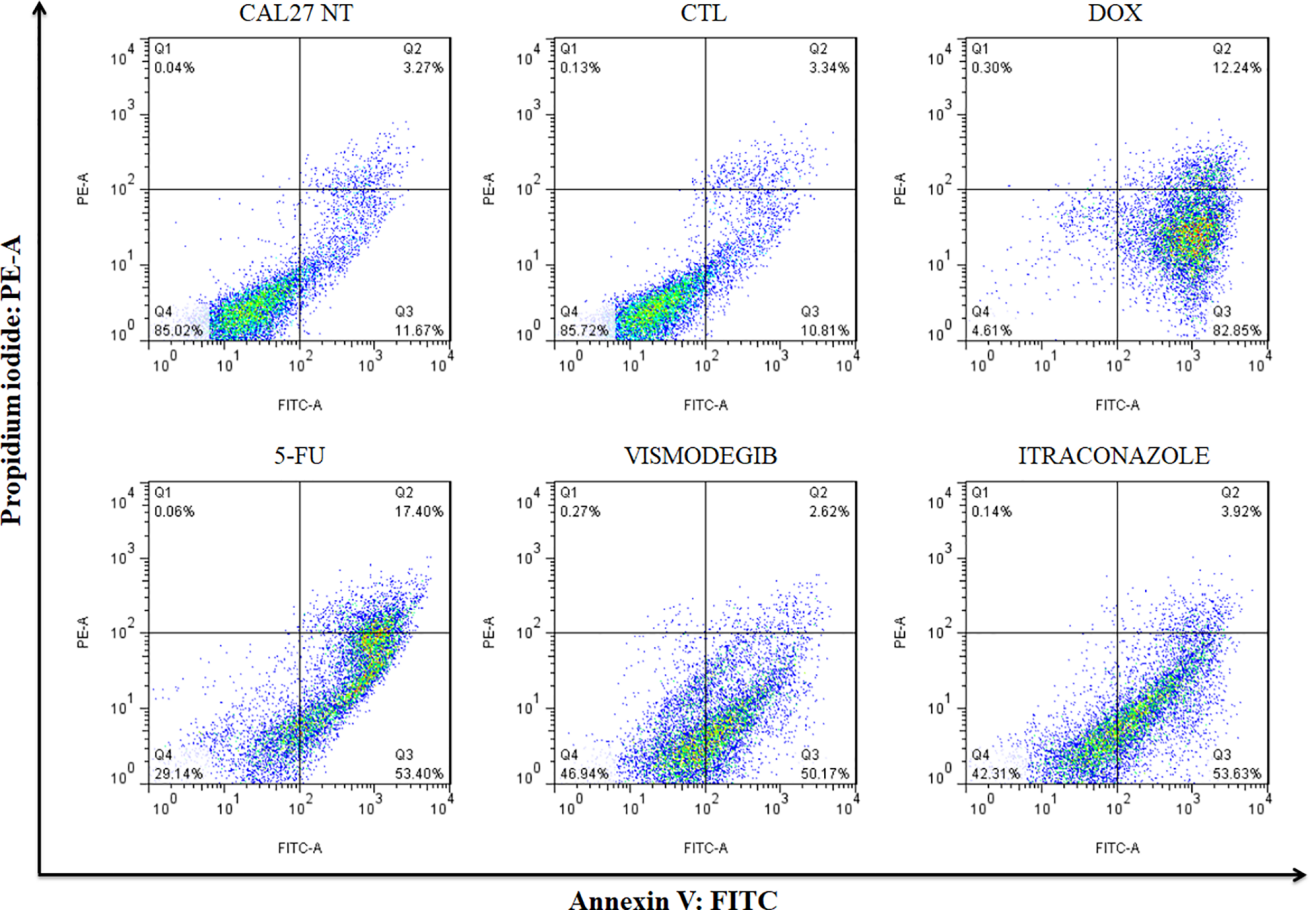
Representative ﬂow cytometry dot plots show the percentage of cells in viable, early apoptotic, late apoptotic, and necrotic stages in CAL27 cells treated with vismodegib and itraconazole after 72 h of treatment. Negative control (DMSO, 0.2%) was used to solubilize and dilute all tested compounds. Doxorubicin (DOX, 1 μg/ml) and 5-FU (10 μg/ml) were used as positive controls. Data represents results from three independent experiments performed in duplicate. Cell debris was omitted from analyses; 10,000 events was analyzed per sample.

After 48 h of treatment with the tested compounds, results did not show a significant increase of cell death by apoptosis. Only after 72 h of treatment with vismodegib or itraconazole, an increase in the number of apoptotic cells was seen in comparison to the negative control and DMSO groups. The positive control groups (doxorubicin and 5-FU) also presented significantly increased populations of apoptotic cells (p < 0.05) (**Figures 4** and **5**).

### SMO Inhibitors Induced DNA Fragmentation

**Figure 6:**
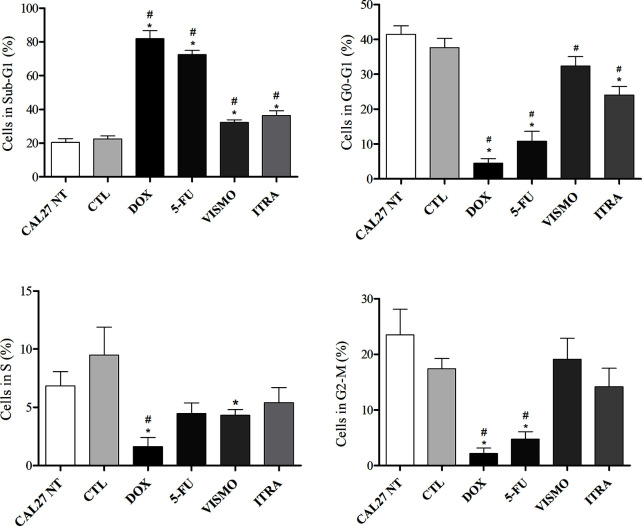
Effects of SMO inhibitors on cell cycle and internucleosomal DNA fragmentation in CAL27 cells after 72 h of treatment. Negative control was treated with the vehicle (0.2% DMSO) used to solubilize and dilute test compounds. Doxorubicin (DOX, 1 μg/ml) and 5-FU (10 μg/ml) were used as positive controls. Values ​​are shown as means ± S.E.M. from three independent experiments carried out in duplicate. Cellular debris was omitted from analyzes and 10,000 events were analyzed per sample. p < 0.05 compared to the CTL group by ANOVA, followed by the Student Newman-Keuls test. * and ^#^ indicate significant differences in relation to negative control (CTL, DMSO 0.2%) and CAL27-NT, respectively.

After 72 h of treatment with vismodegib and itraconazole (50 µg/ml), an increased number of CAL27 cells in the Sub-G1 phase were found compared to negative controls (CAL27 NT and CTL groups), as well as reductions in cell populations in the G0-G1 and S phases (**Figures 6** and **S6**).

## Discussion

HH pathway activity has been shown to lead to the development of several human tumors (16–24, 27, 61), including OSCC (15, 62–65) and other cell lineages of this type of tumor (66). The results presented here corroborate the participation of this signaling pathway in OSCC, providing a basis for efforts to investigate and develop targeted pathway inhibitors. As HH pathway represents a potential pharmacological target in OSCC, some drugs developed to target SMO may prove useful. More than 50 compounds have been identified as inhibitors of HH signaling in cancer (67), and, of these, vismodegib was approved in 2012 by FDA for the treatment of advanced or metastatic adult basal-cell carcinoma. Although vismodegib and itraconazole have been shown to strongly inhibit HH signaling in several types of cancers (34, 53), the inhibition potential in oral cancer cells has not been well investigated.

The present study demonstrates that SMO inhibitors, vismodegib and itraconazole, successfully reduced the gene expression levels of SMO, PTCH1, and GLI1, reduced the viability of OSCC CAL27 cells and altered the morphology of this cell line in a time- and concentration-dependent manner. In addition, these inhibitors induced apoptosis and increased DNA fragmentation, demonstrating the therapeutic potential of inhibiting the expression of HH pathway components in OSCC.

The treatment of CAL27 cells with vismodegib or itraconazole reduced SMO, PTCH1, and GLI1 gene expression after 24 h, similarly to what was observed by Hu et al. (49), who reported reduced HH pathway component expression after treatment with itraconazole and vismodegib (31, 34, 35, 45, 68). GLI1, a transcription factor of HH pathway, is believed to regulate the expression of genes involved in proliferation, survival, and cell viability (69, 70) and the blockage of HH pathway may inhibit these cancer hallmarks.

In fact, after 72 h of treatment with vismodegib or itraconazole, alterations in cell cycle distribution and increases in DNA fragmentation were observed, which seems to indicate apoptosis (71). In addition, treatment with vismodegib was observed to reduce the relative quantity of S-phase cells. Other authors showed that treatment with 50 μM vismodegib led to an increase in the apoptotic fraction in colorectal (72) and prostate cancer cells (73), whereas itraconazole also induced apoptosis in gastric cancer cells (49). On the other hand, cyclopamine, another SMO inhibitor, failed to induce significant apoptosis in human pancreatic carcinoma cells (74).

Therefore, SMO inhibitors tested here were shown to inhibit the growth of OSCC cells through the reduction of cell viability, induction of apoptosis, and alterations of cell cycle phases. Moreover, we found reduced size and increased granularity in CAL27 OSCC cells treated with these SMO inhibitors, which indicates the involvement of apoptosis or senescence either of which cause cells to lose their proliferative potential. This effect has been observed in the use of chemotherapeutic agents, and may represent and interesting marker for the selection of new drugs with anticancer potential (40).

**Figure 7:**
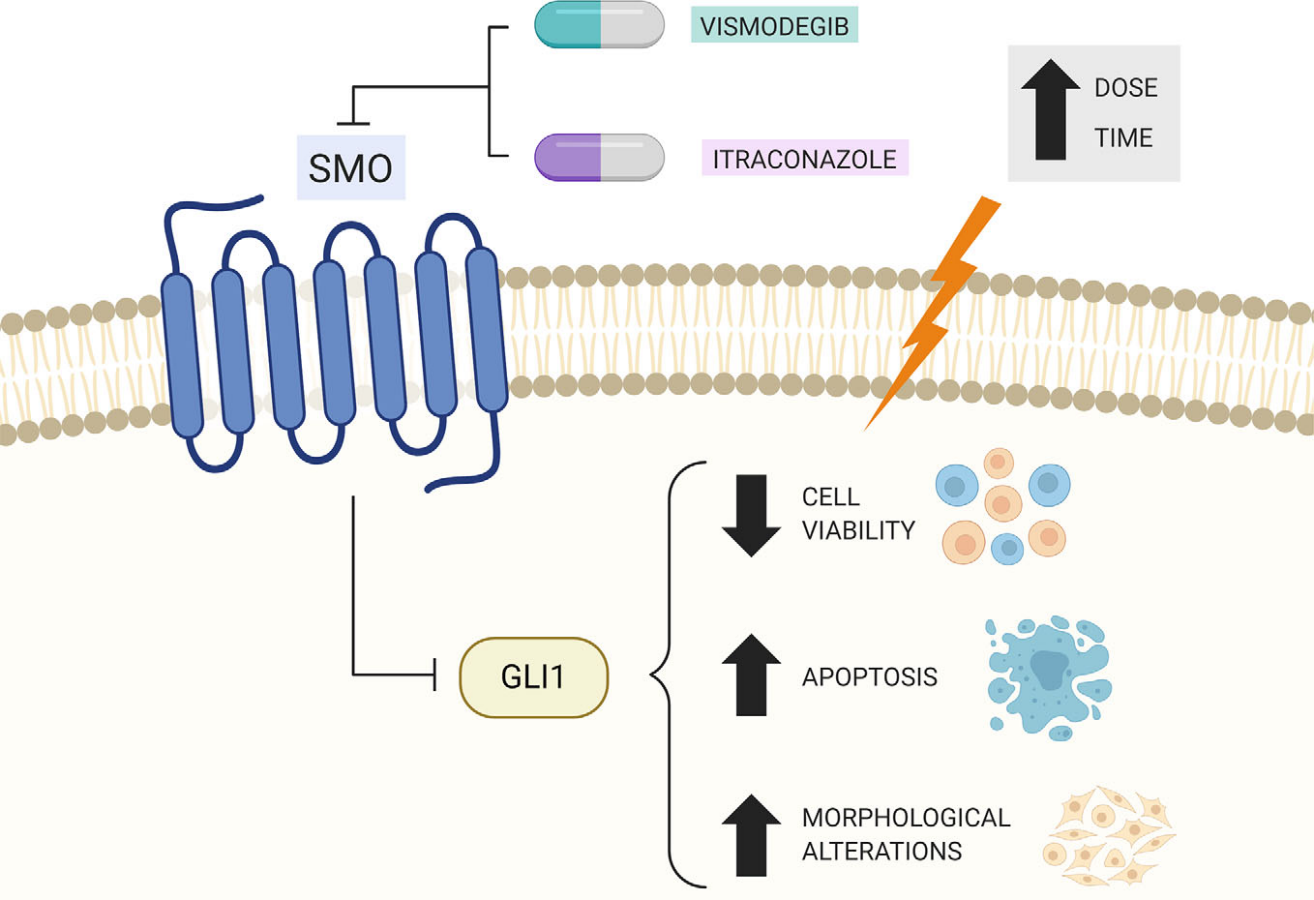
Summary of therapeutic effects of Vismodegib and Itraconazole treatment in CAL27 cells. Created with BioRender.com.

Considering these results, we can indicate that HH pathway is active in OSCC cells and the pharmacological inhibition of this pathway in CAL27 OSCC cell lines with SMO inhibitors reduced the expression of PTCH1, SMO, and GLI1 genes, suggesting a reduction in the activity of this signaling pathway after 24 h of treatment. SMO inhibitors were capable of reducing viability and altering CAL27 OSCC cell line morphology in a time and concentration-dependent manner. In addition, these inhibitors have induced apoptosis and increased DNA fragmentation in this cell type. **Figure 7** summarizes our results.

While the *in vitro* results of the present study are promising in the CAL27 OSCC cell line, with demonstrated effects on viability, morphology, HH gene expression, cell cycle, and cell death, the efficacy of vismodegib and itraconazole in OSCC should be evaluated *in vivo* in the future. This data will be highly relevant in pre-clinical studies designed to assess the potential therapeutic use of vismodegib and itraconazole in OSCC treatments.

## Data Availability Statement

The original contributions presented in the study are included in the article/**Supplementary Material**. Further inquiries can be directed to the corresponding author.

## Ethics Statement

This study was approved by The Institutional Review Board of the Oswaldo Cruz Foundation (FIOCRUZ,Salvador, Bahia, Brazil). All participants signed a term of informed consent to participate in the study.

## Author Contributions

Conceptualization: CR, TP, and DB. Methodology: CR, RD, RF, RC, CS, TP. Software: RG, RD, LV, MV. Validation: RF, LR, RC, MV, LV. Formal analysis: CR, CS, MS, DB, RC, MR. Investigation: RF, RD, MV, LV, RG, AD, CS, and LR. Resources: CR, RC, MR, MS, DB, and AD. Writing—original draft preparation: CR, RF, RD, TP, and MS. Writing—review and editing: CR, RF, RD, MS. Visualization: all authors. Supervision: CR, DB, and RD. Project administration: CR, DB, MR, MS. Funding acquisition: CR. All authors contributed to the article and approved the submitted version.

## Funding

This research was funded by FAPESB, grant number APP006/2016 and National Council for Scientific and Technological Development (CNPq) grant number 308595/2016-5.

## Conflict of Interest

The authors declare that the research was conducted in the absence of any commercial or financial relationships that could be construed as a potential conflict of interest.
