# Supplementary figures and images for: Inhibition of CAL27 Oral Squamous Carcinoma Cell by Targeting Hedgehog Pathway With Vismodegib or Itraconazole
Source: Front Oncol. 2020 Nov 10;10:563838. doi: 10.3389/fonc.2020.563838 (PMC7703359; doi:10.3389/fonc.2020.563838)

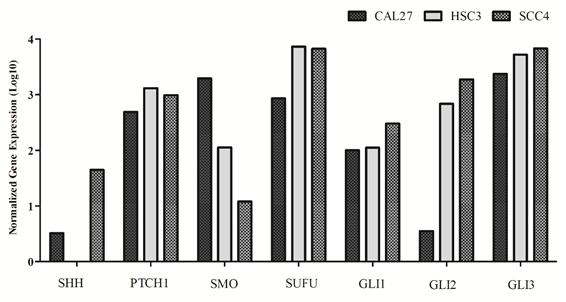

Supplement: Supplementary file 1 [file Image_1.tif]

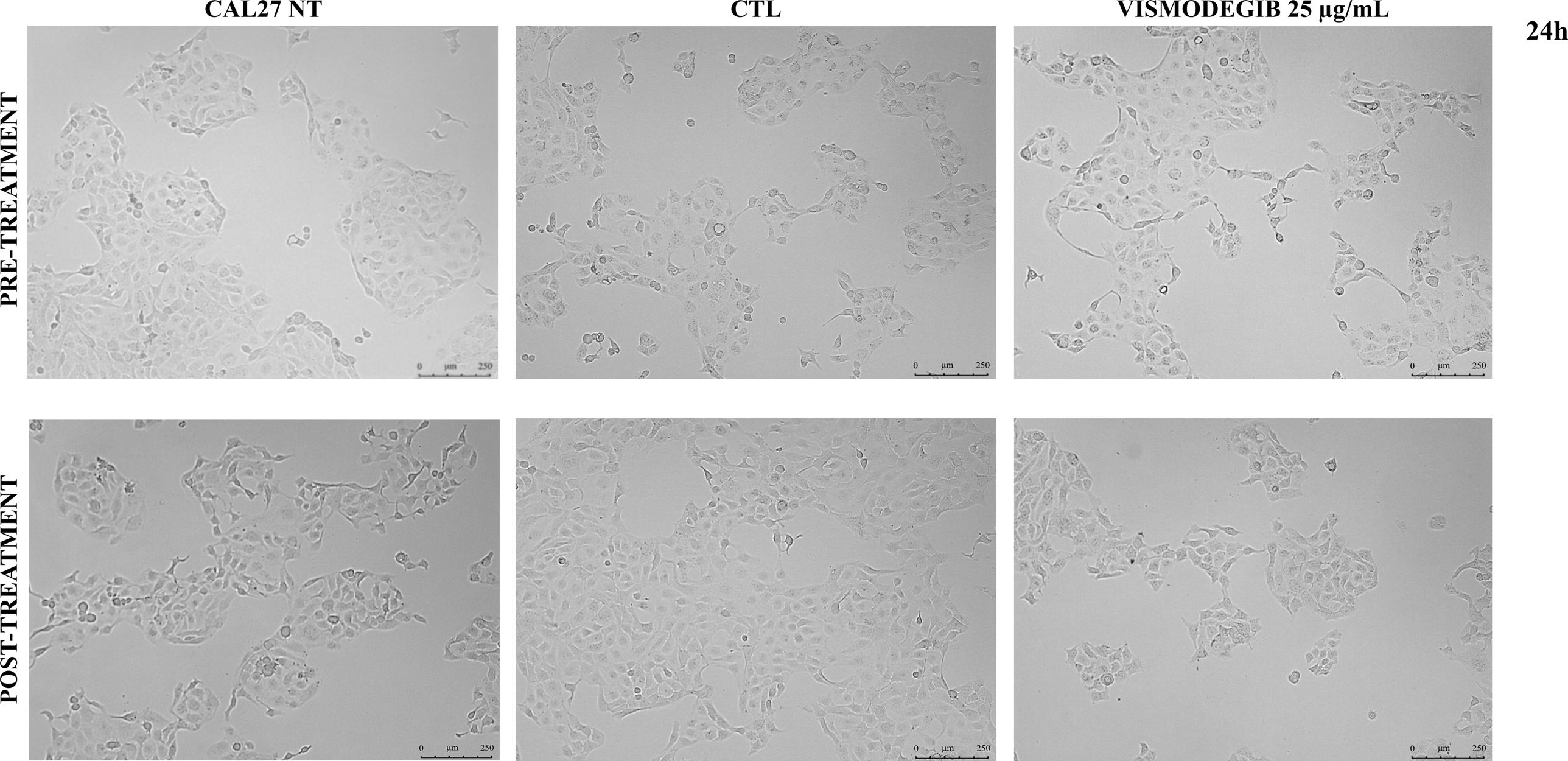

Supplement: Supplementary file 2 [file Image_2.tif]

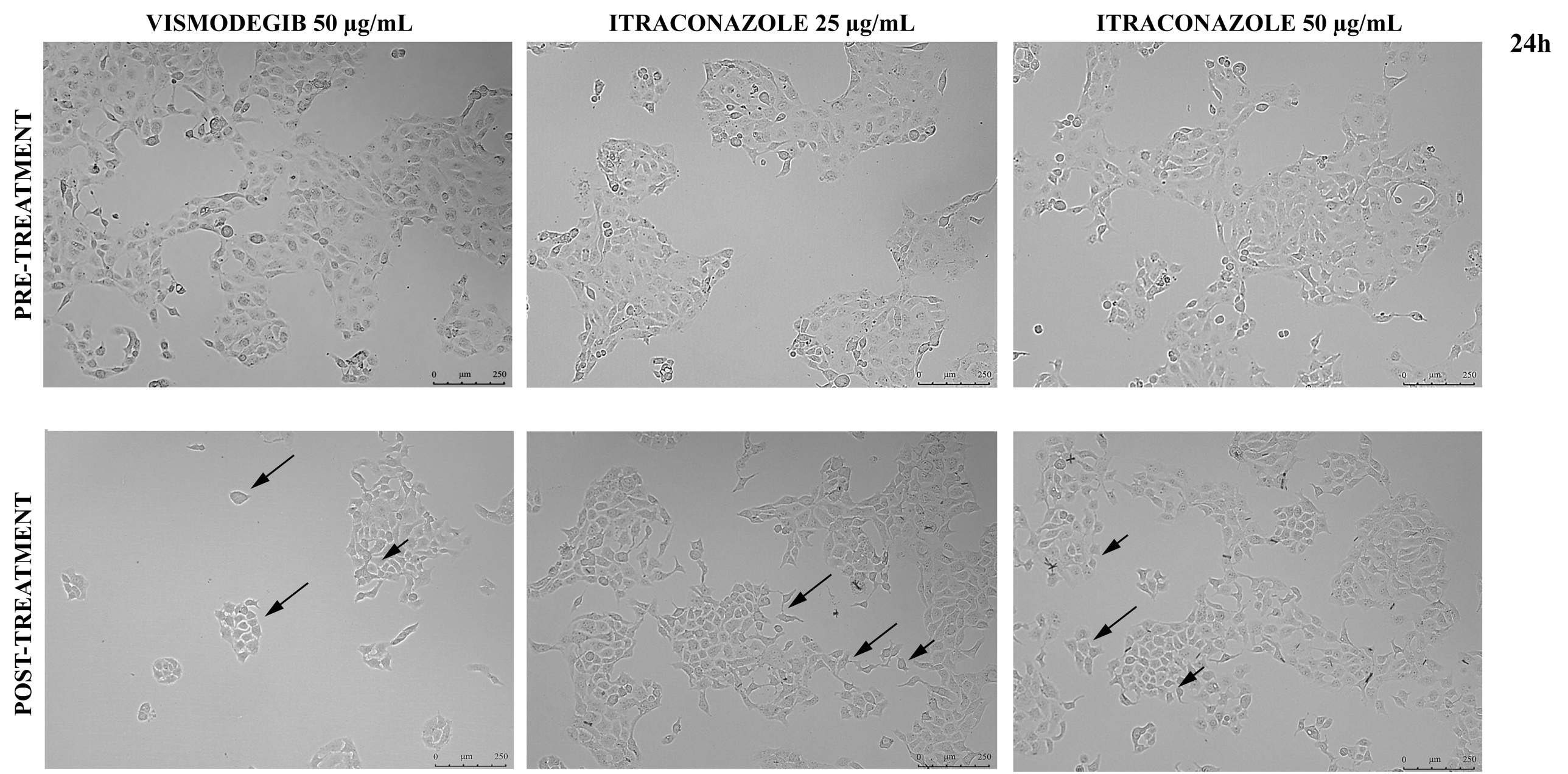

Supplement: Supplementary file 3 [file Image_3.tif]

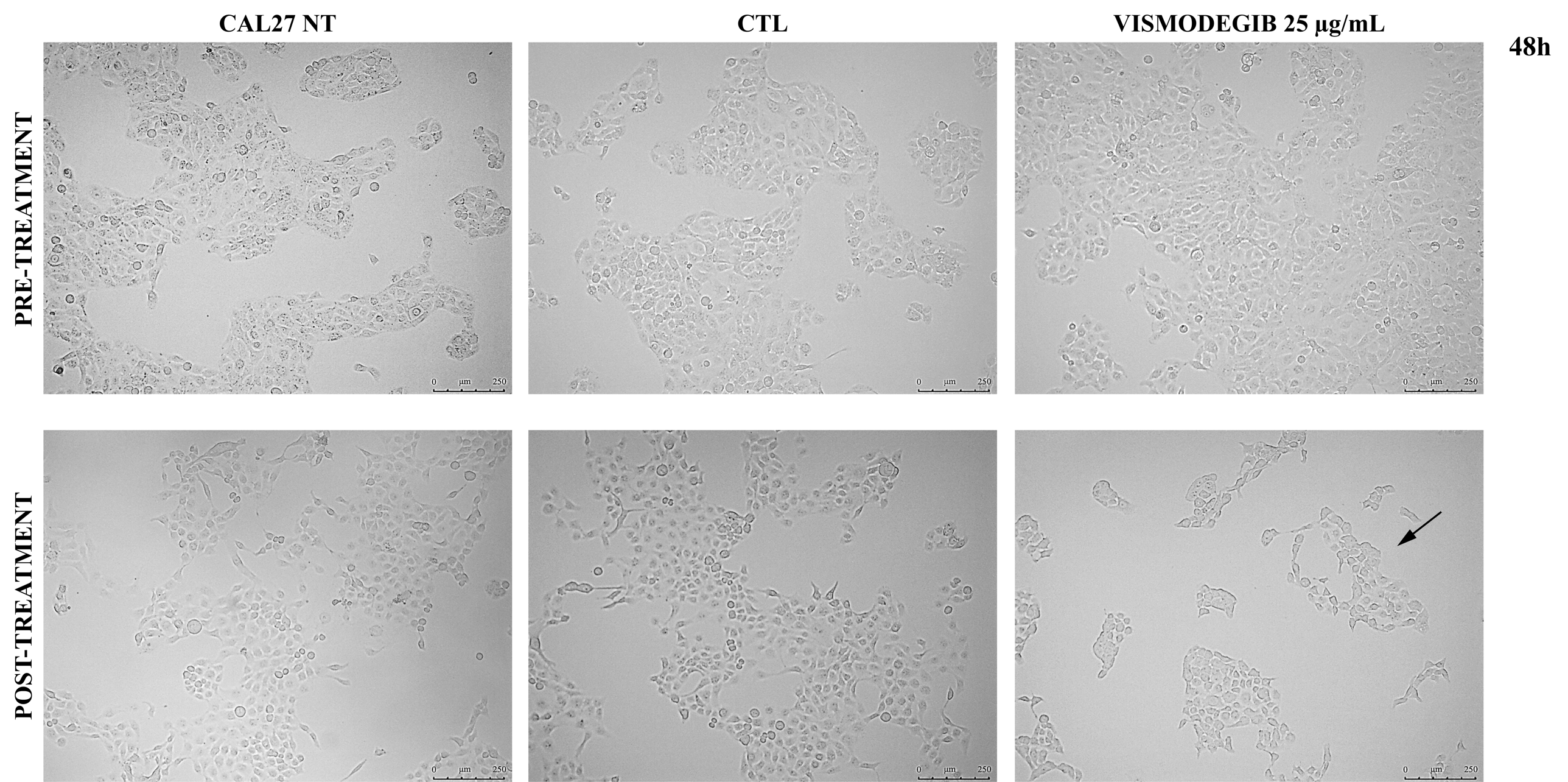

Supplement: Supplementary file 4 [file Image_4.tif]

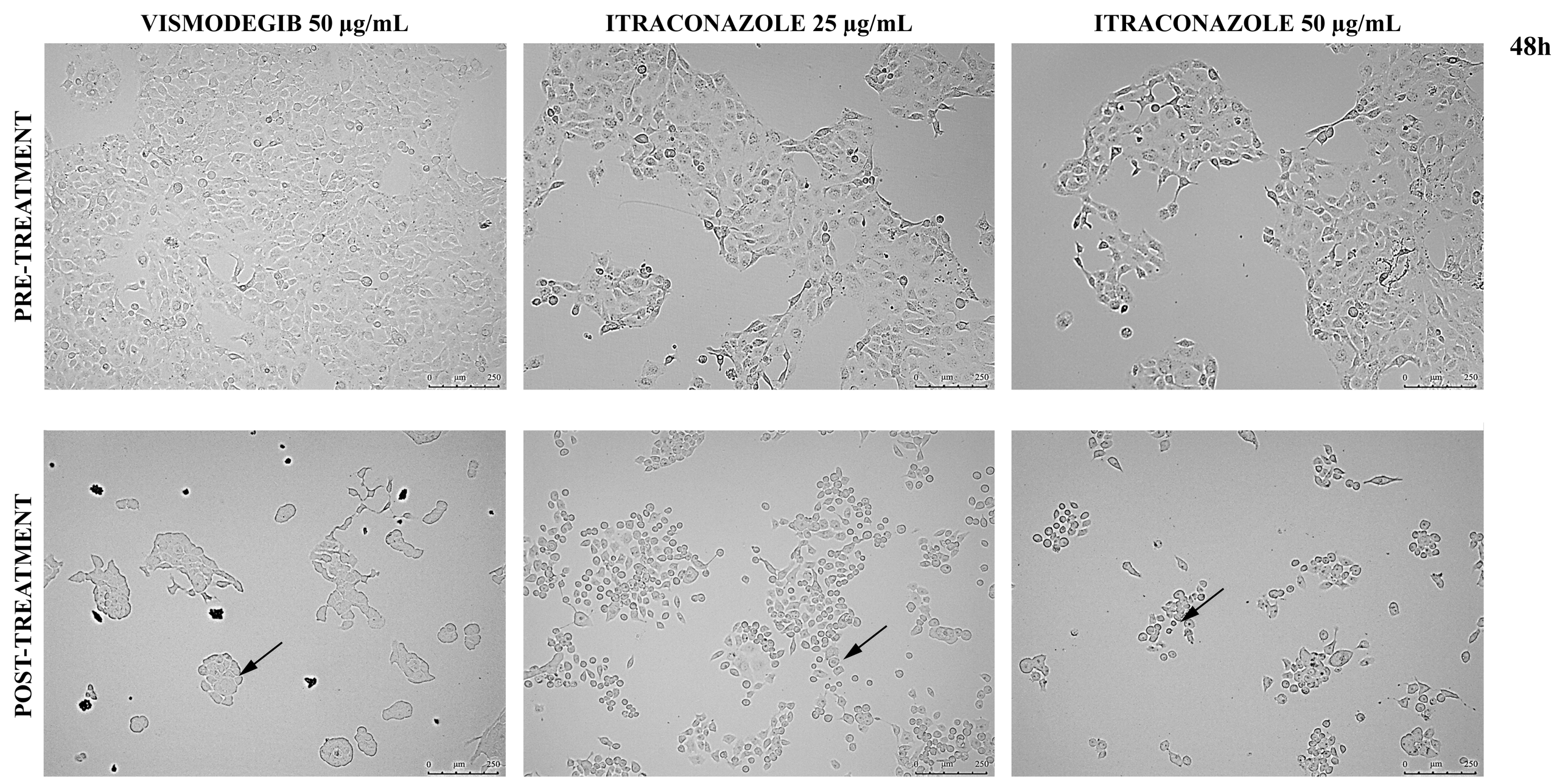

Supplement: Supplementary file 5 [file Image_5.tif]

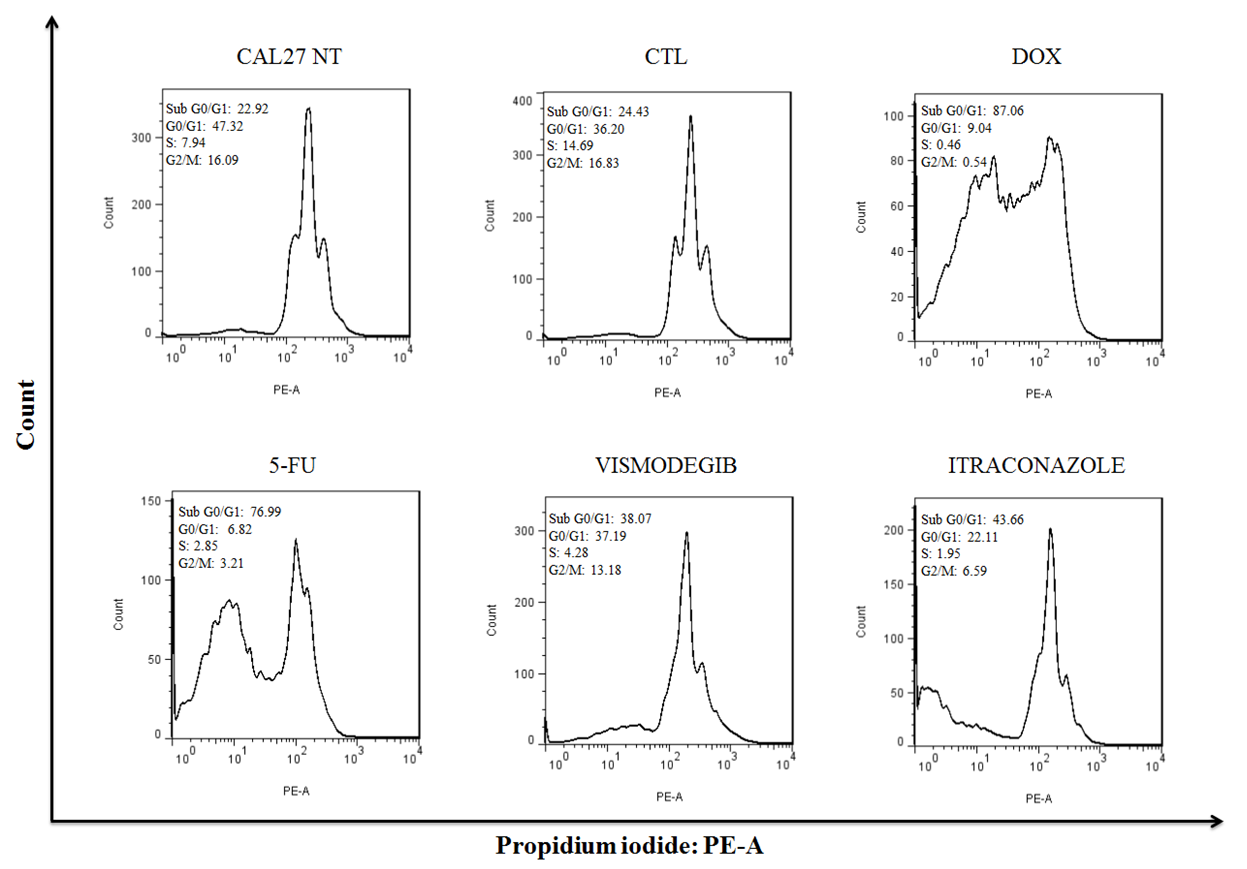

Supplement: Supplementary file 6 [file Image_6.tif]
