# Supplementary material for: Inhibition of CAL27 Oral Squamous Carcinoma Cell by Targeting Hedgehog Pathway With Vismodegib or Itraconazole
Source: Front Oncol. 2020 Nov 10;10:563838. doi: 10.3389/fonc.2020.563838 (PMC7703359; doi:10.3389/fonc.2020.563838)
Supplement: Supplementary file 7 [file Table_1.docx]

Supplementary Material

**Supplementary Table 1.** Human cell lineages used for in vitro experimentation

| **Cell lineage** | **Histological type** | | **Source** |
| --- | --- | --- | --- |
| CAL27 | Oral squamous cell carcinoma | Antoine Lacassagne Center  Bio-Oncology Laboratory  Nice - France | |
| HSC3 | Oral squamous cell carcinoma | Japan Health Sciences Research Resource Bank | |
| SCC4 | Oral squamous cell carcinoma | Sidney Farber Cancer Institute, Cell Growth and Regulation Division and Department of Physiology, Harvard Medical School | |
