# Supplementary material for: Inhibition of CAL27 Oral Squamous Carcinoma Cell by Targeting Hedgehog Pathway With Vismodegib or Itraconazole
Source: Front Oncol. 2020 Nov 10;10:563838. doi: 10.3389/fonc.2020.563838 (PMC7703359; doi:10.3389/fonc.2020.563838)
Supplement: Supplementary file 8 [file Table_2.docx]

Supplementary Material

**Supplementary Table 2.** Inhibitor concentrations as determined for selected assays

| **Assay** | **Itraconazole** | **Vismodegib** | **Doxorubicin** | **5-FU** |
| --- | --- | --- | --- | --- |
|  | **Concentration (μg/mL)** | | | |
| **Cytotoxicity activity - Alamar Blue** | 0.19 - 50 | 0.19 - 50 | 0.19-25 | 0.19-25 |
| **Cellular viability -TrypanBlue** | 25 and 50 | 25 and 50 | - | - |
| **Gene expression** | 25 and 50 | 25 and 50 | 1 | 10 |
| **Cell cycle** | 50 | 50 | 1 | 10 |
| **Cell death (Annexin V/PI)** | 50 | 50 | 1 | 10 |
